# Diffusible Factors Secreted by Glioblastoma and Medulloblastoma Cells Induce Oxidative Stress in Bystander Neural Stem Progenitors

**DOI:** 10.1177/1759091416662808

**Published:** 2016-08-09

**Authors:** Neha Sharma, Nicholas W. Colangelo, Sonia M. de Toledo, Edouard I. Azzam

**Affiliations:** 1 Department of Radiology, New Jersey Medical School-Cancer Center, Rutgers University, NJ, USA

**Keywords:** brain tumors, neural stem progenitors, reactive oxygen species, ionizing radiation or bystander effect, intercellular communication

## Abstract

Harmful effects that alter the homeostasis of neural stem or progenitor cells (NSPs) can affect regenerative processes in the central nervous system. We investigated the effect of soluble factors secreted by control or ^137^Cs-γ-irradiated glioblastoma or medulloblastoma cells on redox-modulated endpoints in recipient human NSPs. Growth medium harvested from the nonirradiated brain tumor cells, following 24 h of growth, induced prominent oxidative stress in recipient NSPs as judged by overall increases in mitochondrial superoxide radical levels (*p* < .001), activation of c-jun *N*-terminal kinase, and decrease in the active form of FoxO3a. The induced oxidative stress was associated with phosphorylation of p53 on serine 15, a marker of DNA damage, induction of the cyclin-cyclin dependent kinase inhibitors p21^Waf1^ and p27^Kip1^, and perturbations in cell cycle progression (*p* < .001). These changes were also associated with increased apoptosis as determined by enhanced annexin V staining (*p* < .001) and caspase 8 activation (*p* < .05) and altered expression of critical regulators of self-renewal, proliferation, and differentiation. Exposure of the tumor cells to radiation only slightly altered the induced oxidative changes in the bystander NSPs, except for medium from irradiated medulloblastoma cells that was more potent at inducing apoptosis in the NSPs than medium from nonirradiated cells (*p* < .001). The elucidation of such stressful bystander effects provides avenues to understand the biochemical events underlying the development or exacerbation of degenerative outcomes associated with brain cancers. It is also relevant to tissue culture protocols whereby growth medium conditioned by tumor cells is often used to support the growth of stem cells.

## Introduction

Neural stem cells are uncommitted, multipotent cells generated throughout the life of an individual. They exist in embryonic, fetal, and adult tissues and give rise to rapidly amplifying progenitors responsible for proper formation and functioning of the central nervous system (Gage and Temple, 2013). Therefore, signaling events that affect their survival or self-renewal and differentiation will likely impact development of the central nervous system and may result in the emergence or exacerbation of progressive degenerative outcomes. Ischemia, traumatic brain injury, exposure to ionizing radiation, and cancer are among the insults or pathological conditions that can impinge on the integrity of neural stem cells (Limoli et al., 2004, 2007; Chen et al., 2014; Baulch et al., 2015). Whereas there is a rapidly growing literature on the direct effects of harmful agents (e.g., ischemia, ionizing radiation) on stem cells, the influence of cues propagated from tumor cells on the integrity of neural stem or progenitor cells (NSPs) within or near their niche remains mostly unknown (Ivanov and Hei, 2014). A better understanding of these effects, particularly following exposure of nearby tumors to therapeutic agents, is particularly important because harmful changes that perturb the homeostasis of nonirradiated bystander NSPs can lead to cognitive, motor, and behavioral impairments, and the emergence of new cancer (Monje and Palmer, 2003).

Glioblastoma multiforme is the most common malignant brain tumor in adults (Minniti et al., 2009). It is an extremely aggressive cancer associated with neurologic deterioration (Boele et al., 2015). In contrast, medulloblastoma is the most common pediatric malignant brain tumor. It is located in the cerebellum, which is crucial for motor function and has been implicated in higher level cognition (Ramnani, 2006). Surgery, chemotherapy, and radiotherapy are used to treat patients afflicted with either of these tumors. In contrast to the poor survival of patients treated for glioblastoma (5-year median survival below 10%; Delgado-Lopez and Corrales-Garcia, 2016), the 5-year survival rate for children with medulloblastoma is 73% (Smith et al., 2010); however, the latter suffer from debilitating conditions and a high rate of secondary malignancies (Friedman et al., 2010). Therefore, understanding the responses of NSPs neighboring the disease site, and recipient of cues modulated by therapeutic agents such as ionizing radiation, promises to be enlightening due to the regenerative potential of NSPs.

Tumor cells secrete in their native environment systemic factors that include soluble molecules such as cytokines, growth factors, and also toxic metabolites (Charles et al., 2011). These factors can interact with cells within their range (astrocytes, microglia, and NSPs; Lorger, 2012) leading to important biological changes in the interacting cells. Furthermore, exposure to therapeutic radiation can alter the nature of the secreted molecules or their amounts, further modulating the induced biological changes. In this study, we examine the effects of secreted elements on NSPs using the medium transfer strategy (Mothersill and Seymour, 2004). Notably, the spread of harmful effects from irradiated to nonirradiated cells in the vicinity (i.e., bystander effects) was observed following *in vitro* and *in vivo* exposures to ionizing radiation (Azzam et al., 2003; Shao et al., 2003; Matsumoto et al., 2007; Prise and O'Sullivan, 2009; Hei et al., 2011). In this study, we have investigated the induction of oxidative stress, perturbations in cell cycle progression, DNA damage, survival, and altered expression of critical regulators (e.g., FoxO3a, Sox2, and Bmi1) in NSPs recipient of growth medium harvested from glioblastoma or medulloblastoma cells before and after exposure to cesium-137 γ rays. The rationale was to test the hypothesis that essential physiological functions in NSPs are significantly affected when they are present within or in close proximity to malignant neoplasms, and that the induced changes are enhanced when the tumor cells are irradiated.

## Materials and Methods

### Neural Stem Cells

Human H9 neural stem cells were from GIBCO®/Life Technologies (Carlsbad, CA). They were derived from the NIH approved H9 (WA09) embryonic stem cells. Here, we use the term NSPs to refer to all classes of immature cells derived from H9 cells that may be present in the population under study. The H9 NSPs were grown in StemPro® NSC SFM medium consisting of KnockOut™ D-MEM/F-12 with StemPro® Neural Supplement (2%), epidermal growth factor (20 ng/mL), basic fibroblast growth factor (bFGF, 20 ng/mL), and GlutaMAX™-I (2 mM) as adherent cultures in flasks precoated with CELLStart™ (GIBCO®/Life Technologies). The cells were seeded at a density of 50,000 cells per cm^2^. To help maintain the cells in an undifferentiated state, the medium was supplemented daily with bFGF (10 ng/mL). The cells were fed every 2 days, and upon reaching 90% confluency, they were passaged at a ratio of 1:2 using StemPro® Accutase® (GIBCO®/Life Technologies). They were incubated at 37℃ in a humidified atmosphere of 5% CO_2_ in air.

### Tumor Cells

Human T98G (CRL 1690™) glioblastoma and Daoy (HTB-186™) medulloblastoma cells were from the ATCC (Manassas, VA). They were maintained in MEM supplemented with 10% fetal bovine serum, 100 U/mL penicillin, 100 µg/mL streptomycin (CellGro), and 2 mM glutagro™ (CorningMediatech) at 37℃ in a humidified atmosphere of 5% CO_2_ in air. They were fed every 2 days and were subcultured when they were ∼90% confluent.

### Media Transfer Strategy and Irradiation

T98G and Daoy monolayer cell cultures underwent change of medium to neural stem cell medium when they were ∼70% confluent. They were washed 2 × with D-PBS with calcium and magnesium (PBS^+^), the StemPro® NSC SFM medium was added, and the cultures were incubated for 1 h at 37℃ prior to exposure at 37℃ to a mean absorbed dose of 12 Gy from a ^137^Cs γ-ray source (3 Gy/min; J L Shepherd, Mark I, San Fernando, CA). Following irradiation, the cells were incubated for 24 h.

Near confluent NSPs were subcultured at a 1:2 ratio and seeded in 100 mm in diameter dishes 24 h prior to incubation with conditioned media harvested from control or irradiated tumor cells (designated CCM and ICM, respectively). The CCM and ICM conditioned for 24 h by the tumor cells were harvested, centrifuged to eliminate cellular debris, and added to the NSP cultures. After this point, none of the growth factors were added to the cultures. Following 24 h incubation, the H9 NSPs were harvested, rinsed with PBS^+^, and processed for analyses of biological endpoints.

### Mitochondrial Superoxide Radicals

They were detected using MitoSOX™ Red (ThermoFisher Scientific, Waltham, MA). Briefly, H9 cells grown in 12-well plates were stained with 5 µM MitoSOX™ in StemPro® NSC SFM medium and incubated in the dark at 37℃ for 10 to 15 min. They were then gently dissociated with accutase, washed 2 × with PBS^+^, and analyzed by flow cytometry at an excitation/emission maxima of 510/580 nm.

### Intracellular Reactive Oxygen Species

They were detected using CM-H2DCFDA (5 -(and-6)-chloromethyl-2',7'-dichlorodihydrofluorescein diacetate, acetyl ester), a general oxidative stress indicator (ThermoFisher Scientific). H9 cells grown in 12-well plates were stained with 10 µM CM-H2DCFDA in StemPro® NSC SFM medium and incubated in the dark at 37℃ for 10 to 15 min. The cells were then dissociated with accutase, washed 2× with PBS^+^, and analyzed using flow cytometry at an excitation/emission of 492–495/517–527 nm.

### Mitochondrial Membrane Potential

It was assessed using the cytofluorimetric, lipophilic cationic dye, 5,5′,6,6′-tetrachloro-1,1′,3,3-tetraethylbenzimi-dazolylcarbocyanine iodide (JC-1; Cayman Chemical, Ann Arbor, MI). H9 cells grown in 12-well plates were stained in the absence of light with 200 µL of JC-1 dye diluted 1:10 in StemPro® NSC SFM medium and incubated for 10 to 15 min. They were then gently dissociated with accutase, washed 2× with PBS^+^, and analyzed using flow cytometry at an excitation/emission of 485/535 nm.

### Immunoblotting

Cell fractionation was performed to enrich the nuclear and cytosolic fractions as we have described (Zhang et al., 2012). Proteins in the subcellular fractions were separated by SDS-PAGE and immunoblotted. Anti-FoxO3a, (1:1000, clone 75D8), anti-phospho-p53 (Ser15; 1:1000, cat. no. 9824), anti-phospho-SAPK/JNK (Thr183/Tyr185; 1:1000, cat. no.9251), anti-SAPK/JNK (1:1000, cat. no.9252), anti-Sox2 (1:1000, clone L73B4, cat. no. 4195), and anti-Bmi1 (1:1000, cat. No. 2830) antibodies were from Cell Signaling Technology. Anti-survivin (1:1000, cat. no. 24479, Abcam), anti-p21^Waf1/Cip1^ (1:1000 cat. no. 05-345, Millipore), and anti-p27^Kip1^ (1:1000, C-19, sc-528, Santa Cruz) antibodies were also used. The antibody dilutions were made in 5% solution of bovine serum albumin in Tris-Buffered Saline and Tween 20 (TBST). Anti-mouse or anti-rabbit IgG secondary antibodies conjugated with horseradish peroxidase were used for *chemiluminescence detection.* Ponceau S Red staining was used as loading control. In the figures, the relative intensity (R.I.) is intensity (I) of a band (z) is normalized against its control (c) and their Ponceau S Red intensities (P): R.I. = [I(z)/P(z)]/[I(c)/P(c)].

### Cell Cycle Analysis

H9 cells were washed 2× with PBS^+^, dissociated with accutase, washed with ice-cold PBS^+^, fixed with chilled 80% ethanol, and left at −20℃ overnight. They were then washed 2× with cold PBS^+^, stained with propidium iodide (PI) (50 µg/mL), treated with RNase A (10 mg/mL) for 1 h, and analyzed by flow cytometry.

### Detection of Apoptosis

H9 cells were subjected to Annexin V (ApopNexin Annexin V FITC Apoptosis Kit, Millipore, Billerica, MA) and caspase 8 (Vybrant® FAM Caspase-8 Assay Kit, Molecular probes) assays according to the manufacturer’s instructions. The FLICA-stained cells were analyzed by flow cytometry at 488 nm excitation.

### Statistics

Experiments were repeated at least three times. The results are represented as the average of the values obtained in independent experiments ± SEM. For cell cycle analysis, measurement of apoptosis by the Annexin V assay, and mitochondrial depolarization by staining with JC-1, changes in cell proportions in response to the different experimental conditions were tested by the χ^2^ test, using the Holm-Sidak test to control for multiple comparisons (Holm, 1979; Aickin and Gensler, 1996; Glantz, 2005) and the Yates correction for 2 × 2 contingency tables. For measurements of mitochondrial superoxide radicals, intracellular reactive oxygen species (ROS), and apoptosis by Caspase 8, median fluorescence intensity values in the different experimental conditions were averaged over at least three experiments and compared using a one-way ANOVA, with the Tukey test to control for multiple comparisons. For analysis by χ^2^, overall significance was tested using an R × C contingency table before performing the post hoc Holm-Sidak test for individual comparisons using 2 × 2 contingency tables. For ANOVA, the *F*-statistic was used to test the overall significance of the data set before performing the post hoc Tukey test for individual comparisons. For all experiments, overall significance for a family of comparisons was set at *p* < .05.

## Results

### Incubation of Bystander NSPs in Growth Medium From Control or γ-Irradiated Brain Tumor Cells Upregulates the Level of Mitochondrial Superoxide Radicals

**Figure 1.**
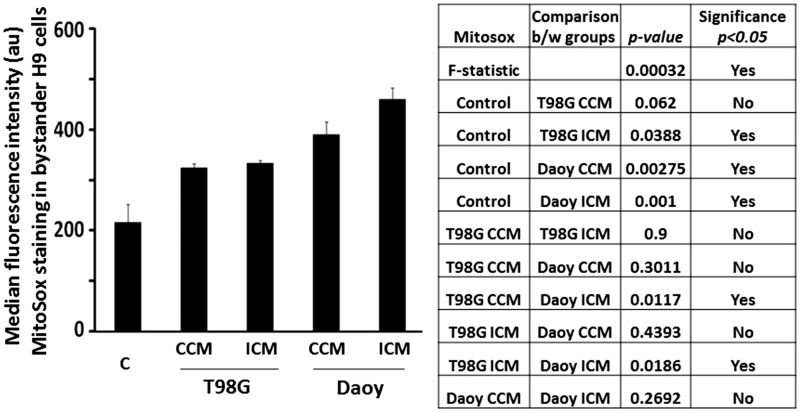
Factors secreted by brain tumor cells modulate superoxide levels in mitochondria of bystander NSPs. H9 NSPs cultured for 24 h in medium harvested from control (CCM) or irradiated (ICM) T98G (glioblastoma) or Daoy (medulloblastoma) cell cultures were subjected to MitoSox analysis using flow cytometry. Bar graph represents average median fluorescence intensity in arbitrary units (au) from three independent experiments ± SEM. Values in the different experimental conditions were compared using a one-way ANOVA, with the Tukey test to control for multiple comparisons.

To shed light on the drivers of debilitating outcomes associated with brain cancer, we investigated the impact of extracellular secretion by glioblastoma and medulloblastoma cells on mitochondrial ROS levels in NSPs: Mitochondrial ROS play multiple roles in signaling cascades and have a role in pathological conditions. Here, we assessed mitochondrial superoxide radicals (${\text{O}}_{2}^{•-}$) by increased fluorescence intensity of NSPs incubated with MitoSOX Red dye that specifically targets to mitochondria in live cells. Relative to control, incubation of H9 NSPs for 24 h, in medium (CCM) in which nonirradiated T98G glioblastoma cells were grown for 24 h, resulted in a 1.5-fold increase, but the increase was not statistically significant (1.5-fold change, *p* = .062; *n* = 3; Figure 1). However, incubation of the NSPs in medium conditioned for 24 h by T98G cells exposed to 12 Gy of γ rays (ICM) did result in a significant increase relative to control (1.5-fold change *p* = .039, *n* = 3), but the increase was not significantly different relative to CCM (1.0-fold change, *p* = .9, *n* = 3; Figure 1). Similarly, increases in mitochondrial ${\text{O}}_{2}^{•-}$ were observed in H9 cells cultured for 24 h in CCM (2.0-fold change, *p* = .003; *n* = 3) and ICM (1.75-fold change, *p* = .001; *n* = 3) from Daoy medulloblastoma cells (Figure 1), but the CCM and ICM were not significantly different from one another (1.2-fold change, *p* = .269, *n* = 3). The effects of the CCM from the Daoy cells were not significantly different from the effects of the CCM (1.2-fold change, *p* = .301, *n* = 3) and ICM (1.2-fold change, *p* = .440, *n* = 3) of the T98G cells. However, the mitochondrial ${\text{O}}_{2}^{•-}$ levels in H9 cells receiving ICM from Daoy cells were significantly increased relative to that of T98G CCM (1.4-fold, *p* = .012, *n* = 3) and ICM (1.4-fold, *p* = .019, *n* = 3; Figure 1). Therefore, diffusible factor(s) secreted by nonirradiated or irradiated glioblastoma or medulloblastoma cells lead to biochemical events that result in enhanced levels of oxidative stress in bystander NSPs. Surprisingly, the effects induced by the irradiated medulloblastoma cells were greater than those induced by factors released by the T98G glioblastoma cells.

### Stress Signals Propagated From Brain Tumor Cells Lead to Loss of Mitochondrial Membrane Potential, Increase in Intracellular ROS, and JNK Activation in Bystander NSPs

**Figure 2.**
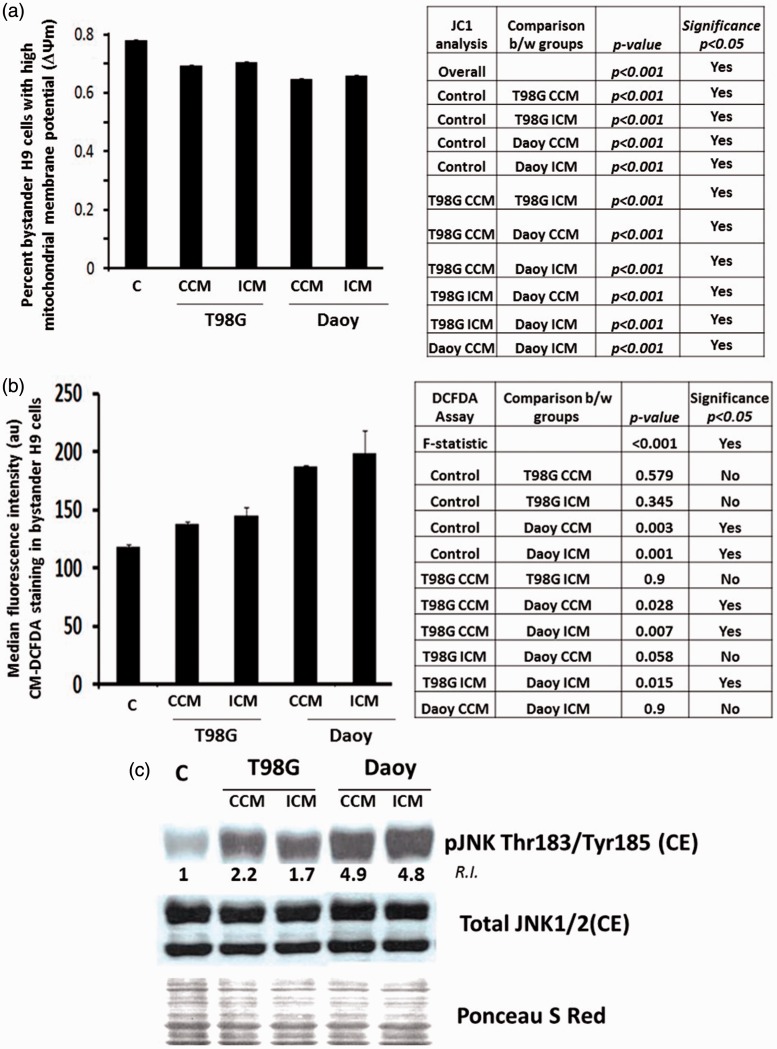
Factors secreted by brain tumor cells alter mitochondrial membrane potential (ΔΨm), upregulate intracellular ROS levels, and activate redox-modulated kinase in bystander NSPs. (a) H9 NSPs cultured for 24 h with medium conditioned for 24 h by control (CCM) or irradiated (ICM) T98G or Daoy brain tumor cells were subjected to ΔΨm analysis using JC-1 probe by flow cytometry. Bar graph is average of three independent experiments ± SEM of NSPs with high ΔΨm. (b) H9 NSPs were treated as in (a) and subjected to analyses of intracellular ROS using CM-DCFDA assay by flow cytometry. Bar graph represents average of median fluorescence intensity in arbitrary units (au) ± SEM (*n* = 3). (c) H9 NSPs were treated as in (a) and subjected to immunoblot analysis (*n* = 3). One way ANOVA was used in analyses of the results in panel 2 A and χ^2^ test was used in analysis of the results in panel 2B as detailed in Materials and Methods section.

Excess mitochondrial ${\text{O}}_{2}^{•-}$ levels can initiate a cascade of reactions leading to the generation of other forms of ROS (Chuaqui and Petkau, 1987), which can be even more detrimental to functioning of mitochondria. A side effect is depolarization of the mitochondrial membrane, resulting in an increase in intracellular ROS. These ROS may react with macromolecules in their vicinity affecting signaling pathways essential for healthy survival. Consistent with the increase in mitochondrial superoxide (Figure 1), H9 NSPs cultured for 24 h in CCM from T98G showed an 8.6% decrease (*p* < .001; *n* = 3) in the fraction of NSPs with highly polarized mitochondria as assessed by the JC-1 probe relative to control, while the ICM from T98G showed a 7.64% decrease (*p* < .001; *n* = 3) relative to control (Figure 2(a)). Similarly, H9 cells incubated in CCM from Daoy cells resulted in a 13% decrease (*p* < .001; *n* = 3), and those NSPs receiving ICM from Daoy cells resulted in a 12% decrease (*p* < .001; *n* = 3; Figure 2(a)). For both the T98G cells (*p* = .012; *n* = 3) and Daoy cells (*p = *.008; *n* = 3), the NSPs had a 1% higher fraction of cells with highly polarized mitochondria when incubated in the ICM relative to the CCM. Relative to the T98G CCM, the Daoy CCM induced a 4.6% decrease (*p* < .001; *n* = 3) and the Daoy ICM induced a 3.5% decrease (*p* < .001; *n* = 3). Relative to the T98G ICM, the Daoy CCM caused a 5.6% decrease (*p* < .001; *n* = 3) and the Daoy ICM a 4.5% decrease (*p* < .001; *n* = 3; Figure 2(a)). Therefore, extracellular secretion by brain tumor cells impacts an important parameter of mitochondrial functionality.

As predicted, the decrease in mitochondrial membrane potential (Figure 2(a)) was associated with an increase in intracellular ROS in H9 cells, as revealed by the CM*-*H2DCFDA assay (Figure 2(b)). Relative to control, 1.6- and 1.7-fold respective increases in the median fluorescence intensity, reflecting ROS levels, were observed when H9 cells were cultured in CCM (*p* < .003; *n* = 3) or ICM (*p* < .001; *n* = 3) from Daoy cells. However, H9 cells cultured in CCM (*p* = .58; *n* = 3) or ICM (*p* = .35; *n* = 3) from T98G cells showed no significant increases relative to control nor relative to one another (*p* = .9; *n* = 3; Figure 2(b)). The CCM from Daoy cells brought a significant 1.4-fold increase relative to CCM (*p* = .028; *n* = 3) of T98G cells, but its 1.3-fold increase over the ICM of T98G cells did not meet significance (*p* = .058; *n* = 3). The ICM from Daoy cells resulted in 1.4-fold increases in intracellular ROS relative to both T98G CCM (*p* = .007; *n* = 3) and T98G ICM (*p* = .015; *n* = 3; Figure 2(b)).

The c-jun *N*-terminal kinase (c-JNK), a member of the mitogen activated protein kinase family (MAPK), is activated through cytoplasmic phosphorylation events by an increase in cellular oxidative stress (McCubrey et al., 2006). Activated JNK affects proliferation, apoptosis, and other cellular processes (Chen et al., 1996; Zhang and Liu, 2002). Consistent with the increase in ROS levels (Figures 1 and 2(b)), immunoblot analyses revealed activation of JNK in the cytoplasmic fraction of H9 cells (Figure 2(c)). Relative to control, H9 NSPs cultured in CCM or ICM from T98G cells exhibited 2.2- and 1.7-fold respective increases in p-JNK (Thr183/Tyr185) levels (*n* = 3). Similarly, 4.9- and 4.8-fold respective increases in p-JNK (Thr183/Tyr185) were observed in H9 cells cultured in CCM and ICM from Daoy cells (*n* = 3). These changes were due to posttranslational modifications and did not involve changes in the basal level of JNK (Figure 2(c)). These results support the induction of redox-activated stress kinase in bystander H9 cells, recipient of diffusible factors from brain tumor cells.

### Factors Secreted by Brain Tumors Lead to Altered Expression of Regulatory Proteins Involved in ROS Detoxification in Bystander NSPs

**Figure 3.**
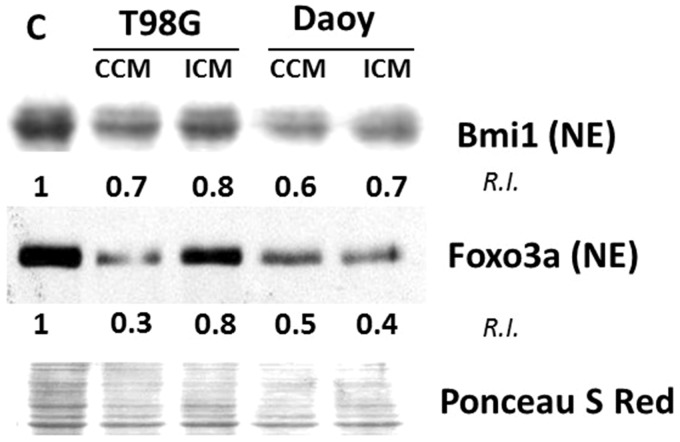
Factors secreted by brain tumor cells alter expression of regulatory genes involved in ROS detoxification in bystander NSPs. H9 NSPs cultured for 24 h with medium conditioned for 24 h by control (CCM) or irradiated (ICM) T98G or Daoy cell cultures were subjected to immunoblot analysis of lysates enriched in nuclear extract (NE) proteins (*n* = 3). The relative intensity (R.I.) is intensity (I) of a band (z) normalized against its control (c) and respective Ponceau S Red intensity (P). R.I. = [I(z)/P(z)]/[I(c)/P(c)].

Bmi1, a member of the polycomb group of proteins, provides resistance to oxidative stress and promotes survival by regulating mitochondrial dynamics, suggesting negative regulation of Bmi1 by oxidative stress (Nakamura et al., 2012). Bmi1 has been judged essential for the maintenance of hematopoietic and neural stem cells (Yadirgi et al., 2011). Relative to control, immunoblotting revealed 30% and 20% decrease in Bmi1 levels in nuclear fractions of H9 NSPs, following incubation for 24 h in CCM or ICM from T98G cells, respectively (*n* = 3; Figure 3). Similarly, 40% and 30% respective decreases were detected in H9 cells cultured in CCM and ICM from Daoy cells (*n* = 3; Figure 3).

The FoxO group of transcription factors is another key regulator of adult stem cells with an important role in resistance to oxidative stress (Paik et al., 2009). Analyses of protein lysates, enriched in the nuclear fraction, of H9 cells incubated in CCM or ICM from T98G cells showed 70% and 20% respective decreases, relative to control, in the active form of FoxO3a (*n* = 3, Figure 3). Similarly, H9 cells incubated in CCM or ICM from Daoy cells showed 50% and 60% respective decreases (*n* = 3; Figure 3).

### Diffusible Factors Secreted by Brain Tumors Promote Induction of DNA Damage and Lead to Perturbation in Cell Cycle Progression in Bystander NSPs

**Figure 4.**
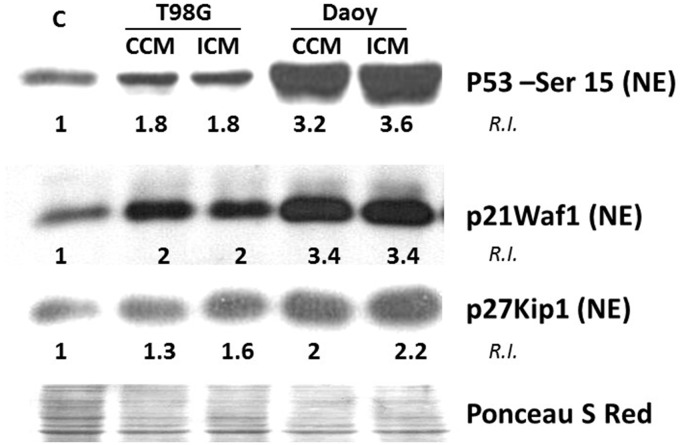
Propagation of oxidative stress from brain tumor cells is associated with induction of DNA damage responsive genes in bystander NSPs. H9 NSPs cultured for 24 h in medium conditioned for 24 h by control (CCM) or irradiated (ICM) T98G or Daoy brain tumor cells were subjected to immunoblot analysis in lysates enriched in the nuclear fraction (*n* = 3). The relative intensity (R.I.) is intensity (I) of a band (z) normalized against its control (c) and respective Ponceau S Red intensity (P). R.I. = [I(z)/P(z)]/[I(c)/P(c)].

An increase in oxidative stress leads to DNA damage and activation of an array of DNA damage responsive signaling pathways (Georgakilas et al., 2014). Prime among the signaling events is activation of ATM kinase followed by phosphorylation of p53 (Kastan and Lim, 2000). Phosphorylation of p53 by ATM on serine-15 residue is a marker of DNA damage (Canman et al., 1998). The immunoblot results in Figure 4 indicate a 1.8-fold increase over control in P-p53-Ser15 levels in H9 cells cultured, respectively, in CCM and ICM from T98G cells (*n* = 3). H9 cells cultured in CCM or ICM from Daoy cells showed 3.2- and 3.6-fold increases, respectively (*n* = 3).

Consistent with activation of ATM/p53 signaling, an increase in levels of the downstream effector p21^Waf1^ was detected (Figure 4). Relative to control, H9 cells cultured in CCM or ICM from T98G cells exhibited 2-fold increase in p21^Waf1^ levels (*n* = 3). Similarly, CCM or ICM from Daoy cells resulted in a 3.4-fold increases (*n* = 3). The p21^Waf1^ protein is a universal inhibitor of cyclin-dependent kinases that regulate transition from G_1_ to S phase in the cell cycle (Elledge, 1996). Upon exposure to oxidizing agents, up-regulation of p21^Waf1^ mediates the stress-induced G_1_ checkpoint. In addition to the increase in p21^Waf1^, an increase in p27^Kip1^, an inhibitor of cyclin D/cdk4 and cyclin E/cdk2, was also detected. We observed a 1.3- and 1.6-fold increase in p27^Kip1^ level in H9 cells incubated, respectively, in CCM and ICM from T98G cells and a 2- and 2.2-fold increase when the cells were incubated in CCM and ICM from Daoy cells.

**Figure 5.**
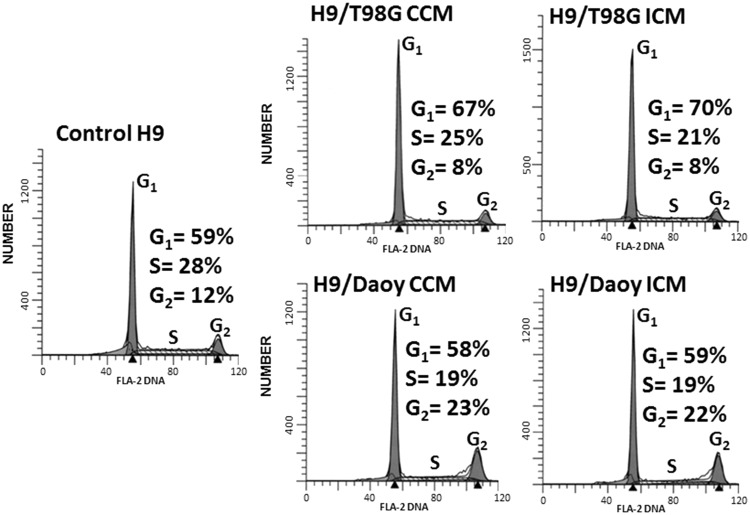
Propagation of oxidative stress from brain tumor cells to bystander NSPs is associated with perturbation in cell cycle progression. H9 NSPs cultured for 24 h in medium conditioned for 24 h by T98G or Daoy cells were subjected to cell cycle analysis by flow cytometry. Events (10,000) were collected for analysis and representative histograms are shown (ordinates are number of cells and abscissa is DNA content as assessed by propidium iodide staining; *n* = 3).

Consistent with up-regulation of p21^Waf1^, the percentage of H9 NSPs in G_1_ phase in cultures, incubated for 24 h in CCM or ICM from T98G cells, increased relative to control (7% and 11% respective increases in the representative experiment shown, *p* < .001; *n* = 3; Figure 5). Relative to the CCM, the ICM from T98G cells resulted in a 3% increase (*p* < .001; *n* = 3) in the proportion of NSPs in G_1_. However, the CCM and ICM from Daoy cells did not significantly alter the proportion of NSPs in G_1_ (*p* = .4; *n* = 3). In contrast, at 24 h, a significant fraction of H9 cells accumulated in G_2_ phase when cultured for 24 h in CCM or ICM from Daoy cells (11% and 9% respective increases in representative experiment shown, *p* < .001; *n* = 3; Figure 5), with a corresponding decrease in the S phase of the cell cycle (9% and 10% respective decreases in representative experiment shown, *p* < .001). For the CCM and ICM of T98G cells, the G2 phase had significant decreases (4% and 4% respectively, *p* < .001) as did the S phase (4% and 7%, respectively, *p* < .001). These results suggest a differential effect being induced by the two tumor cell types. Together, the data support the role of oxidative stress in inducing DNA damage and perturbing cell cycle progression in bystander NSPs recipient of diffusible factors from brain tumor cells.

### Brain Tumor Cells Transmit Signals That Decrease Survival of Bystander H9 NSPs

**Figure 6.**
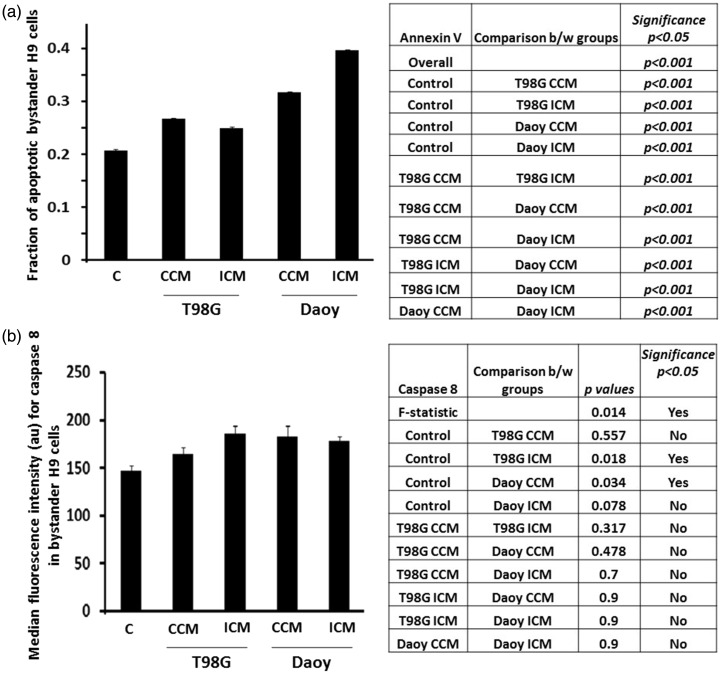
Factors secreted by brain tumor cells promote apoptotic death in bystander NSPs. (a) H9 NSPs cultured for 24 h in medium conditioned for 24 h by T98G or Daoy cells were subjected to Annexin V/PI assay and analyzed by flow cytometry. Events (10,000) were collected for the analysis and changes in cell proportions in response to the different experimental conditions were tested by the χ^2^ test, using the Holm-Sidak test to control for multiple comparisons. Significance was set at *p < .05*. Bar graph represents average of four independent experiments ± SEM. (b) H9 cells were treated as in (a) and subjected for caspase 8 assay by flow cytometry. Bar graph represents average median fluorescence intensity in arbitrary units (au) from five independent experiments ± SEM. The average values for each treatment were compared using a one-way ANOVA, with the Tukey test to control for multiple comparisons.

A likely outcome of excess oxidative stress and elevated DNA damage is reduced cellular viability (Azzam et al., 2012). Annexin V/PI staining can identify cells in apoptosis by positive Annexin V staining. Analyses of Annexin V/PI staining (Figure 6(a)) showed that incubation of H9 NSPs for 24 h in CCM or ICM from T98G cells results in 6% and 4% respective increases, relative to control, in the fraction of apoptotic cells (*p* < .001; *n* = 4). Incubation of H9 cells in CCM or ICM from Daoy cells resulted in 11% and 19% increases (*p* < .001; *n* = 4; Figure 6(a)). Relative to the effects of T98G cell CCM on the NSPs, the ICM from T98G cells resulted in a 2% decrease (*p* < .001; *n* = 4) in apoptotic cells. The ICM from Daoy cells, however, caused an 8% increase (*p* < .001; *n* = 4) relative to CCM from Daoy cells. Relative to the T98G cell CCM, the Daoy CCM resulted in a 5% increase (*p* < .001; *n* = 4) and the Daoy ICM in a 13% increase (*p* < .001; *n* = 4). Relative to the T98G ICM, the Daoy CCM caused a 7% increase (*p* < .001; *n* = 4) and the Daoy ICM a 15% increase (*p* < .001; *n* = 4).

Caspase 8 is the initiator caspase that propagates the apoptosis signal by direct cleavage of downstream effector caspases and other proteins. Our flow cytometry analyses in H9 NSPs incubated for 24 h in ICM from T98G or CCM from Daoy cells showed 27% and 24% increases in median fluorescence intensity for activated caspase 8, relative to control (*p* = .02 and *p* = .03, respectively; *n* = 5; Figure 6(b)). The other comparisons for caspase 8 did not reach significance.

**Figure 7.**
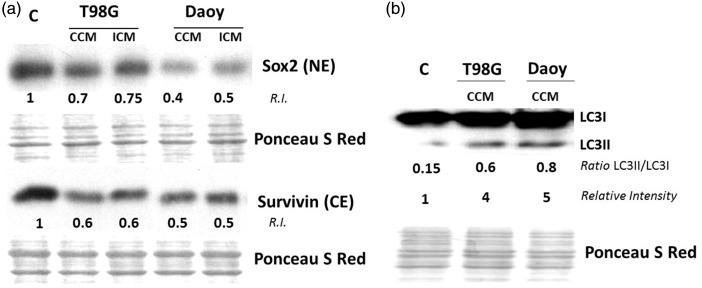
Factors secreted by brain tumor cells alter levels of survival regulators in bystander NSPs. (a) H9 NSPs cultured for 24 h in medium conditioned for 24 h by T98G or Daoy cells were subjected to immunoblot analysis in lysates enriched in either cytoplasmic (CE) or nuclear fractions (NE) (*n* = 3). (b) H9 NSPs were treated as in (a) and analysed by immunoblotting for LC3I lipidation to LC3II (*n* = 3). The relative intensity (R.I.) is intensity (I) of a band (z) normalized against its control (c) and respective Ponceau S Red intensity (P). R.I. = [I(z)/P(z)]/[I(c)/P(c)].

The increase in cell death (Figure 6(a)) prompted us to investigate modulation of critical regulators of survival. Sox2 is important for the maintenance of progenitor characteristics. Its inactivation in mice causes embryonic lethality as a result of decreased proliferation and increased apoptosis (Miyagi et al., 2008). In contrast, survivin protects cells from programmed cell death (Fukuda and Pelus, 2006). Feng et al. (2013) demonstrated the critical role of Sox2 in protecting NSPs from apoptosis by regulating *SURVIVIN* gene expression and inhibiting apoptosis. Relative to control, immunoblot analyses showed that H9 cells cultured in CCM or ICM from T98G cells experienced 30% and 25% decline in Sox2 levels, and ∼40% decrease in survivin levels, respectively (*n* = 3; Figure 7(a)). Similarly, H9 cells cultured in CCM or ICM from Daoy cells showed 60% and 50% decrease in Sox2 levels and 50% decrease in survivin levels, respectively (*n* = 3; Figure 7(a)).

Autophagy is induced by elevated levels of ROS (Filomeni et al., 2015). Consistent with induction of autophagy in H9 cells, immunoblot analyses revealed an increase in LC3I (cytosolic form) lipidation to LC3II (membrane bound form associated with autophagic vesicle formation) in H9 cells cultured in medium conditioned by brain tumor cells. Relative to control, 4- and 5-fold increases in LC3II/LC3I ratio were detected when H9 cells were incubated in CCM from T98G or Daoy cells, respectively (Figure 7(b)). These results suggest that stress defensive mechanisms operate in NSPs present in the tumor niche, which may affect their fate.

## Discussion

Neural stem cells are present throughout the life of an individual. They have the potential to self-renew and differentiate into multiple lineages, and their regenerative power is under intense investigation (Temple, 2001). Therefore, perturbations that threaten their homeostatic integrity may affect brain structure and function. Here, we have investigated the impact of diffusible factors communicated from nonirradiated glioblastoma and medulloblastoma cells on redox-modulated biological endpoints in bystander NSPs. The modulation of the induced changes when the secreted factors are shed by the cancer cells following irradiation was also examined. The results show that prominent oxidative stress associated with DNA damage and cell death is induced in bystander NSPs incubated in medium conditioned by brain tumor cells. Together, the observed biochemical and cellular changes (Figures 1–7) contribute to understanding the basis of secondary pathologies associated with brain cancer before and after radiotherapy. They are specifically relevant to tissue culture protocols wherein CCM or ICM is used to promote the growth of stem cells (Mothersill and Seymour, 2004; Blyth and Sykes, 2011). This study strongly indicates that caution should be exercised when interpreting results of experiments adopting such protocols. Incubation of H9 NSPs in CCM from T98G or Daoy cells results in an increase in mitochondrial superoxide anions, general cellular ROS, activation of stress-responsive kinases, perturbations in cell cycle progression, DNA damage, and apoptosis.

Recently, there has been an emphasis on the relevance of bystander effects in normal tissue toxicities induced following cancer radiotherapy (Newhauser and Durante, 2011). Understanding such toxicities and their underlying mechanisms is particularly pertinent in case of children: With recent advances in early detection and treatment, the probability of long-term survival has increased (Newhauser and Durante, 2011). Unfortunately, toxicities associated with cancer treatments have a significant negative impact on the quality of life (Azzam et al., 2013). This study shows that toxic events can be propagated from brain tumor cells to NSPs. Surprisingly, for most of the endpoints tested, the effects induced in NSPs incubated in ICM from T98G or Daoy cells were only slightly greater than those induced by CCM. This suggests that the level of oxidative stress propagated by these cancer cells to the bystander cells is so high that it is not significantly enhanced following exposure of the tumor cells to ionizing radiation. Dilution of the factors secreted by the tumor cells or their redox modification during media transfer may have contributed to the lack of additional significant effects of the ICM. In this respect, adoption of an expanded time course would be informative as factors with different signaling properties may be secreted as a function of time after irradiation. Further, by 24 h following irradiation, secreted factors with additional toxic effects than those in the CCM may have decayed. The implementation of alternate culture strategies whereby the tumor cells and the bystander NSPs are in intimate co-culture (Domoguaer et al., in press) would be further enlightening; under such conditions, the NSPs would be in immediate and constant interaction with the factors secreted by the tumor cells. Alternatively, the radiation-induced bystander effects may be communicated to the NSPs and other cells in the tumor microenvironment via other modes of intercellular communication, including junctional communication. Notably, our studies (not shown) indicate that stressful events continue to arise in the progeny of NSPs incubated in CCM or ICM and results in a decrease in their survival or proliferative potential, which may be an adaptive response to eliminate the NSPs that harbor damaging changes from the overall stem cell population. Survival of stem cells that harbor damaging effects may have profound implications for long-term health risks (Acharya et al., 2010; Chen et al., 2014).

This study shows that diffusible factors secreted by brain tumor cells has a role in controlling the expression of critical regulators of ROS in bystander NSPs. H9 cells recipient of CCM or ICM from glioblastoma or medulloblastoma cells showed small but consistent relative decreases in the levels of active nuclear FoxO3a. The FoxO transcription factors have been implicated in providing stem cells with resistance to oxidative stress and in maintaining their quiescence. Ablation of FoxO1/3/4 transcription factors resulted in an accumulation of ROS in hematopoietic stem cells concomitant with increased apoptosis (Tothova et al., 2007). In NSPs, the FoxOs regulate diverse physiological processes, including proliferation, resistance to oxidative stress, energy metabolism, and apoptosis (Renault et al., 2009). The decrease in FoxO3a level (Figure 3) is therefore consistent with the enhanced oxidative stress, DNA damage, and apoptosis observed in this study.

Bmi1 has been implicated in cell survival by regulating mitochondrial dynamics and ROS level (Nakamura et al., 2012). Over-expression or activation of mitogen-activated kinases (MAPK) results in phosphorylation of Bmi1 and its dissociation from chromatin thereby de-repressing the target genes (Voncken et al., 2005). H9 cells recipient of CCM or ICM from tumor cells resulted in activation of JNK (Figure 3(c)), a MAPK. In this context, it would be of interest to investigate the role of JNK on the association of Bmi1 with chromatin and impact on mitochondrial functioning in NSPs. The decrease that we observed in Bmi1 levels was not associated with changes in p16^Ink4a^ and p19^Arf^ (not shown), rather it was associated with robust induction of p21^Waf1^ and p27^Kip1^. As would be expected, the latter increases in these cyclin-dependent kinase inhibitors were accompanied with perturbations in cell cycle progression in H9 cells recipient of CCM and ICM from the tumor cells (Figure 5). A significant increase in the fraction of cells accumulating in G_1_ phase was detected in NSPs incubated with CCM or ICM from T98G cells, which may be a defense mechanism providing the NSPs time to repair induced genetic damage prior to DNA replication. Likewise, the accumulation of H9 cells in G_2_ phase upon incubation in CCM or ICM from Daoy cells may provide the NSPs with time to repair DNA damage prior to segregation of the replicated chromosomes in daughter cells (Figure 5). Whether the observed cell cycle checkpoints are transient or persistent would be informative for understanding long-term effects.

Interestingly, p21^Waf1^ has been shown to regulate physiological levels of Sox2 in NSPs by binding to the Sox2 promoter inhibiting transcription of the gene and preventing the promiscuous entry of neural stem cells into S phase (Marques-Torrejon et al., 2013). On the other hand, Bmi1 is involved in NSC proliferation and functions in part by repressing p21^Waf1^ (Subkhankulova et al., 2010). Bmi-1 knockdown resulted in significant decrease in proliferation of progenitors and decrease in survival in embryonic stages (Fasano et al., 2007). Therefore, the decreases in Sox-2 (Figure 7(a)) and Bmi1 (Figure 3) detected in this study are consistent with the observed perturbations in cell cycle progression (Figure 5).

Growing evidence suggests a role for autophagy in maintenance of stem cell pluripotency and self-renewal in response to cellular stress (Filomeni et al., 2015). Dysfunctions in autophagy have been associated with a variety of pathologies including cancer and neurodegenerative diseases (Ghavami et al., 2014). Autophagy serves to clear damaged protein aggregates and impaired organelles, therefore contributing to maintaining cellular homeostasis. It is unclear whether the increased autophagy observed in our studies (Figure 7(b)) constitutes an adaptive response to overcoming the stress propagated from the tumor cells. Further investigation downstream of LC3I lipidation to LC3II in progeny NSPs would be informative.

In sum, this study shows that extracellular secretion by brain tumor cells propagates signaling events that lead to oxidative stress in bystander NSPs. By understanding the molecular events modulated in the process of propagation of bystander effects to NSPs, our understanding of the basis of adverse health effects associated with cancer and its therapies will be enhanced. Experimental approaches using antagonists or inhibitors to the effects of candidate molecules will shed light on ways to manipulate bystander responses toward improved therapeutic outcomes. Notably, using immuno-precipitation, we detected HMGB1 and TGFβ in CCM and ICM from the T98G and Daoy cells used in this study (data not shown), which together with other factors may have contributed to the observed oxidative effects in H9 NSPs.

Expansion of these studies to include a panel of primary early passage glioblastoma and medulloblastoma cells maintained under different cell culture protocols (e.g., as tumor spheres) will further characterize the effect of diffusible factors on oxidative stress in NSPs and enlighten on differential effects that may be induced by tumors derived from different individuals. Tumor cell populations are heterogeneous; their enrichment in a specific cell type (e.g., cancer stem cells) may also affect the magnitude of the bystander effects that they may exert.

## References

[bibr1-1759091416662808] Acharya M. M. Lan M. L. Kan V. H. Patel N. H. Giedzinski E. Tseng B. P. Limoli C. L. (2010) Consequences of ionizing radiation-induced damage in human neural stem cells. Free Radical Biology & Medicine 49: 1846–1855. 20826207 10.1016/j.freeradbiomed.2010.08.021

[bibr2-1759091416662808] Aickin M. Gensler H. (1996) Adjusting for multiple testing when reporting research results: The Bonferroni vs Holm methods. American Journal of Public Health 86: 726–728. 8629727 10.2105/ajph.86.5.726 PMC1380484

[bibr3-1759091416662808] Azzam E. I. De Toledo S. M. Harris A. L. Ivanov V. Zhou H. Amundson S. A. Hei T. K. (2013) The ionizing radiation-induced bystander effect: Evidence, mechanism and significance. In: Sonis S. T. Keefe D. M. (eds) Pathobiology of cancer regimen-related toxicities, New York, NY: Springer, pp. 42–68.

[bibr4-1759091416662808] Azzam E. I. de Toledo S. M. Little J. B. (2003) Oxidative metabolism, gap junctions and the ionizing radiation-induced bystander effect. Oncogene 22: 7050–7057. 14557810 10.1038/sj.onc.1206961

[bibr5-1759091416662808] Azzam E. I. Jay-Gerin J. P. Pain D. (2012) Ionizing radiation-induced metabolic oxidative stress and prolonged cell injury. Cancer Letters 327: 48–60. 22182453 10.1016/j.canlet.2011.12.012 PMC3980444

[bibr6-1759091416662808] Baulch J. E. Craver B. M. Tran K. K. Yu L. Chmielewski N. Allen B. D. Limoli C. L. (2015) Persistent oxidative stress in human neural stem cells exposed to low fluences of charged particles. Redox Biology 5: 24–32. 25800120 10.1016/j.redox.2015.03.001 PMC4371546

[bibr7-1759091416662808] Blyth B. J. Sykes P. J. (2011) Radiation-induced bystander effects: What are they, and how relevant are they to human radiation exposures? Radiation Research 176: 139–157. 21631286 10.1667/rr2548.1

[bibr8-1759091416662808] Boele F. W. Rooney A. G. Grant R. Klein M. (2015) Psychiatric symptoms in glioma patients: From diagnosis to management. Neuropsychiatric Disease and Treatment 11: 1413–1420. 26089669 10.2147/NDT.S65874 PMC4467748

[bibr9-1759091416662808] Canman C. E. Lim D. S. Cimprich K. A. Taya Y. Tamai K. Sakaguchi K. Siliciano J. D. (1998) Activation of the ATM kinase by ionizing radiation and phosphorylation of p53. Science 281: 1677–1679. 9733515 10.1126/science.281.5383.1677

[bibr10-1759091416662808] Charles N. A. Holland E. C. Gilbertson R. Glass R. Kettenmann H. (2011) The brain tumor microenvironment. Glia 59: 1169–1180. 21446047 10.1002/glia.21136

[bibr11-1759091416662808] Chen H. Goodus M. T. De Toledo S. M. Azzam E. I. Levison S. W. Souayah N. (2014) Ionizing radiation perturbs cell cycle progression of neural precursors in the subventricular zone without affecting long-term self-renewal. ASN Neuro 7: 1–16 pii:1759091415578026. . 10.1177/1759091415578026 PMC4461572 26056396

[bibr12-1759091416662808] Chen Y. R. Wang X. Templeton D. Davis R. J. Tan T. H. (1996) The role of c-Jun N-terminal kinase (JNK) in apoptosis induced by ultraviolet C and gamma radiation. Duration of JNK activation may determine cell death and proliferation. The Journal of Biological Chemistry 271: 31929–31936. 8943238 10.1074/jbc.271.50.31929

[bibr13-1759091416662808] Chuaqui C. A. Petkau A. (1987) Chemical reactivity and biological effects of superoxide radicals. Radiation Physics & Chemistry 30: 365–373.

[bibr14-1759091416662808] Delgado-Lopez P. D. Corrales-Garcia E. M. (2016) Survival in glioblastoma: A review on the impact of treatment modalities. Clinical & Translational Oncology: Official Publication of the Federation of Spanish Oncology Societies and of the National Cancer Institute of Mexico. . doi:10.1007/s12094-016-1497-x. 10.1007/s12094-016-1497-x 26960561

[bibr15-1759091416662808] Domoguaer, J., De Toledo, S. M., & Azzam, E. I. (in press). A mimic of the tumor microenvironment: A simple method for generating enriched cell populations and investigating intercellular communication. *Journal of Visualized Experiments*. 10.3791/54429 PMC5092054 27684198

[bibr16-1759091416662808] Elledge S. J. (1996) Cell cycle checkpoints: Preventing an identity crisis. Science 274: 1664–1672. 8939848 10.1126/science.274.5293.1664

[bibr17-1759091416662808] Fasano C. A. Dimos J. T. Ivanova N. B. Lowry N. Lemischka I. R. Temple S. (2007) shRNA knockdown of Bmi-1 reveals a critical role for p21-Rb pathway in NSC self-renewal during development. Cell stem cell 1: 87–99. 18371338 10.1016/j.stem.2007.04.001

[bibr18-1759091416662808] Feng R. Zhou S. Liu Y. Song D. Luan Z. Dai X. Li L. (2013) Sox2 protects neural stem cells from apoptosis via up-regulating survivin expression. The Biochemical Journal 450: 459–468. 23301561 10.1042/BJ20120924

[bibr19-1759091416662808] Filomeni G. De Zio D. Cecconi F. (2015) Oxidative stress and autophagy: The clash between damage and metabolic needs. Cell Death and Differentiation 22: 377–388. 25257172 10.1038/cdd.2014.150 PMC4326572

[bibr20-1759091416662808] Friedman D. L. Whitton J. Leisenring W. Mertens A. C. Hammond S. Stovall M. Neglia J. P. (2010) Subsequent neoplasms in 5-year survivors of childhood cancer: The Childhood Cancer Survivor Study. Journal of National Cancer Institute 102: 1083–1095. 10.1093/jnci/djq238 PMC2907408 20634481

[bibr21-1759091416662808] Fukuda S. Pelus L. M. (2006) Survivin, a cancer target with an emerging role in normal adult tissues. Molecular Cancer Therapeutics 5: 1087–1098. 16731740 10.1158/1535-7163.MCT-05-0375

[bibr22-1759091416662808] Gage F. H. Temple S. (2013) Neural stem cells: Generating and regenerating the brain. Neuron 80: 588–601. 24183012 10.1016/j.neuron.2013.10.037

[bibr23-1759091416662808] Georgakilas A. G. Redon C. E. Ferguson N. F. Kryston T. B. Parekh P. Dickey J. S. Martin O. A. (2014) Systemic DNA damage accumulation under in vivo tumor growth can be inhibited by the antioxidant Tempol. Cancer Letters 353: 248–257. 25069035 10.1016/j.canlet.2014.07.030 PMC4167057

[bibr24-1759091416662808] Ghavami S. Shojaei S. Yeganeh B. Ande S. R. Jangamreddy J. R. Mehrpour M. Los M. J. (2014) Autophagy and apoptosis dysfunction in neurodegenerative disorders. Progress in Neurobiology 112: 24–49. 24211851 10.1016/j.pneurobio.2013.10.004

[bibr25-1759091416662808] Glantz S. A. (2005) Primer of biostatistics, New York, NY: McGraw-Hill Medical Pub.

[bibr26-1759091416662808] Hei T. K. Zhou H. Chai Y. Ponnaiya B. Ivanov V. N. (2011) Radiation induced non-targeted response: Mechanism and potential clinical implications. Current Molecular Pharmacology 4: 96–105. 21143185 10.2174/1874467211104020096 PMC3356574

[bibr27-1759091416662808] Holm S. (1979) A simple sequentially rejective multiple test procedure. Scandinavian Journal of Statistics 6: 65–70.

[bibr28-1759091416662808] Ivanov V. N. Hei T. K. (2014) A role for TRAIL/TRAIL-R2 in radiation-induced apoptosis and radiation-induced bystander response of human neural stem cells. Apoptosis 19: 399–413. 24158598 10.1007/s10495-013-0925-4 PMC3945673

[bibr29-1759091416662808] Kastan M. B. Lim D. S. (2000) The many substrates and functions of ATM. Nature Reviews 1: 179–186. 10.1038/35043058 11252893

[bibr30-1759091416662808] Limoli C. L. Giedzinski E. Baure J. Rola R. Fike J. R. (2007) Redox changes induced in hippocampal precursor cells by heavy ion irradiation. Radiation and Environmental Biophysics 46: 167–172. 17103219 10.1007/s00411-006-0077-9

[bibr31-1759091416662808] Limoli C. L. Giedzinski E. Rola R. Otsuka S. Palmer T. D. Fike J. R. (2004) Radiation response of neural precursor cells: Linking cellular sensitivity to cell cycle checkpoints, apoptosis and oxidative stress. Radiation Research 161: 17–27. 14680400 10.1667/rr3112

[bibr32-1759091416662808] Lorger M. (2012) Tumor microenvironment in the brain. Cancers 4: 218–243. 24213237 10.3390/cancers4010218 PMC3712675

[bibr33-1759091416662808] Marques-Torrejon M. A. Porlan E. Banito A. Gomez-Ibarlucea E. Lopez-Contreras A. J. Fernandez-Capetillo O. Farinas I. (2013) Cyclin-dependent kinase inhibitor p21 controls adult neural stem cell expansion by regulating Sox2 gene expression. Cell Stem Cell 12: 88–100. 23260487 10.1016/j.stem.2012.12.001 PMC3714747

[bibr34-1759091416662808] Matsumoto H. Hamada N. Takahashi A. Kobayashi Y. Ohnishi T. (2007) Vanguards of paradigm shift in radiation biology: Radiation-induced adaptive and bystander responses. Journal of Radiation Research (Tokyo) 48: 97–106. 10.1269/jrr.06090 17327685

[bibr35-1759091416662808] McCubrey J. A. Lahair M. M. Franklin R. A. (2006) Reactive oxygen species-induced activation of the MAP kinase signaling pathways. Antioxidants & Redox Signaling 8: 1775–1789. 16987031 10.1089/ars.2006.8.1775

[bibr36-1759091416662808] Minniti G. Muni R. Lanzetta G. Marchetti P. Enrici R. M. (2009) Chemotherapy for glioblastoma: Current treatment and future perspectives for cytotoxic and targeted agents. Anticancer Research 29: 5171–5184. 20044633

[bibr38-1759091416662808] Miyagi S. Masui S. Niwa H. Saito T. Shimazaki T. Okano H. Okuda A. (2008) Consequence of the loss of Sox2 in the developing brain of the mouse. FEBS Letters 582: 2811–2815. 18638478 10.1016/j.febslet.2008.07.011

[bibr39-1759091416662808] Monje M. L. Palmer T. (2003) Radiation injury and neurogenesis. Current Opinion in Neurology 16: 129–134. 12644738 10.1097/01.wco.0000063772.81810.b7

[bibr40-1759091416662808] Mothersill C. Seymour C. B. (2004) Radiation-induced bystander effects—Implications for cancer. Nature Reviews Cancer 4: 158–164. 14964312 10.1038/nrc1277

[bibr41-1759091416662808] Nakamura S. Oshima M. Yuan J. Saraya A. Miyagi S. Konuma T. Iwama A. (2012) Bmi1 confers resistance to oxidative stress on hematopoietic stem cells. PloS One 7: e36209. 22606246 10.1371/journal.pone.0036209 PMC3350495

[bibr42-1759091416662808] Newhauser W. D. Durante M. (2011) Assessing the risk of second malignancies after modern radiotherapy. Nature Reviews Cancer 11: 438–448. 21593785 10.1038/nrc3069 PMC4101897

[bibr43-1759091416662808] Paik J. H. Ding Z. Narurkar R. Ramkissoon S. Muller F. Kamoun W. S. DePinho R. A. (2009) FoxOs cooperatively regulate diverse pathways governing neural stem cell homeostasis. Cell Stem Cell 5: 540–553. 19896444 10.1016/j.stem.2009.09.013 PMC3285492

[bibr44-1759091416662808] Prise K. M. O'Sullivan J. M. (2009) Radiation-induced bystander signalling in cancer therapy. Nature Reviews Cancer 9: 351–360. 19377507 10.1038/nrc2603 PMC2855954

[bibr101-1759091416662808] Ramnani, N. (2006). The primate cortico-cerebellar system: anatomy and function. *Nature Reviews Neuroscience**7*, 511–522. 10.1038/nrn1953 16791141

[bibr45-1759091416662808] Renault V. M. Rafalski V. A. Morgan A. A. Salih D. A. Brett J. O. Webb A. E. Brunet A. (2009) FoxO3 regulates neural stem cell homeostasis. Cell Stem Cell 5: 527–539. 19896443 10.1016/j.stem.2009.09.014 PMC2775802

[bibr46-1759091416662808] Shao C. Stewart V. Folkard M. Michael B. D. Prise K. M. (2003) Nitric oxide-mediated signaling in the bystander response of individually targeted glioma cells. Cancer Research 63: 8437–8442. 14679007

[bibr47-1759091416662808] Smith M. A. Seibel N. L. Altekruse S. F. Ries L. A. Melbert D. L. O'Leary M. Reaman G. H. (2010) Outcomes for children and adolescents with cancer: Challenges for the twenty-first century. Journal of Clinical Oncology 28: 2625–2634. 20404250 10.1200/JCO.2009.27.0421 PMC2881732

[bibr48-1759091416662808] Subkhankulova T. Zhang X. Leung C. Marino S. (2010) Bmi1 directly represses p21Waf1/Cip1 in Shh-induced proliferation of cerebellar granule cell progenitors. Molecular and Cellular Neuroscience 45: 151–162. 20600931 10.1016/j.mcn.2010.06.006

[bibr49-1759091416662808] Temple S. (2001) The development of neural stem cells. Nature 414: 112–117. 11689956 10.1038/35102174

[bibr50-1759091416662808] Tothova Z. Kollipara R. Huntly B. J. Lee B. H. Castrillon D. H. Cullen D. E. Gilliland D. G. (2007) FoxOs are critical mediators of hematopoietic stem cell resistance to physiologic oxidative stress. Cell 128: 325–339. 17254970 10.1016/j.cell.2007.01.003

[bibr51-1759091416662808] Voncken J. W. Niessen H. Neufeld B. Rennefahrt U. Dahlmans V. Kubben N. Rapp U. R. (2005) MAPKAP kinase 3pK phosphorylates and regulates chromatin association of the polycomb group protein Bmi1. The Journal of Biological Chemistry 280: 5178–5187. 15563468 10.1074/jbc.M407155200

[bibr52-1759091416662808] Yadirgi G. Leinster V. Acquati S. Bhagat H. Shakhova O. Marino S. (2011) Conditional activation of Bmi1 expression regulates self-renewal, apoptosis, and differentiation of neural stem/progenitor cells in vitro and in vivo. Stem Cells 29: 700–712. 21305672 10.1002/stem.614

[bibr53-1759091416662808] Zhang J. de Toledo S. M. Pandey B. N. Guo G. Pain D. Li H. Azzam E. I. (2012) Role of the translationally controlled tumor protein in DNA damage sensing and repair. Proceedings of the National Academy of Sciences of the United States of America 109: E926–E933. 22451927 10.1073/pnas.1106300109 PMC3341051

[bibr54-1759091416662808] Zhang W. Liu H. T. (2002) MAPK signal pathways in the regulation of cell proliferation in mammalian cells. Cell Research 12: 9–18. 11942415 10.1038/sj.cr.7290105

